# Deep learning pathological microscopic features in endemic nasopharyngeal cancer: Prognostic value and protentional role for individual induction chemotherapy

**DOI:** 10.1002/cam4.2802

**Published:** 2019-12-20

**Authors:** Kuiyuan Liu, Weixiong Xia, Mengyun Qiang, Xi Chen, Jia Liu, Xiang Guo, Xing Lv

**Affiliations:** ^1^ State Key Laboratory of Oncology in South China Collaborative Innovation Center for Cancer Medicine Guangzhou Guangdong China; ^2^ Department of nasopharyngeal carcinoma Sun Yat‐sen University Cancer Centre Guangzhou Guangdong China; ^3^ Department of Intensive Care Center Sun Yat‐sen University Cancer Centre Guangzhou Guangdong China

**Keywords:** DeepSurv, induction chemotherapy, nasopharyngeal carcinoma, pathological microfeatures

## Abstract

**Background:**

To explore the prognostic value and the role for treatment decision of pathological microscopic features in patients with nasopharyngeal carcinoma (NPC) using the method of deep learning.

**Methods:**

The pathological microscopic features were extracted using the software QuPath (version 0.1.3. Queen's University) in the training cohort (Guangzhou training cohort, n = 843). We used the neural network DeepSurv to analyze the pathological microscopic features (DSPMF) and then classified patients into high‐risk and low‐risk groups through the time‐dependent receiver operating characteristic (ROC). The prognosis accuracy of the pathological feature was validated in a validation cohort (n = 212). The primary endpoint was progression‐free survival (PFS).

**Results:**

We found 429 pathological microscopic features in the H&E image. Patients with high‐risk scores in the training cohort had shorter 5‐year PFS (HR 10.03, 6.06‐16.61; *P* < .0001). The DSPMF (C‐index: 0.723) had the higher C‐index than the EBV DNA (C‐index: 0.612) copies and the N stage (C‐index: 0.593). Furthermore, induction chemotherapy (ICT) plus concomitant chemoradiotherapy (CCRT) had better 5‐year PFS to those received CCRT (*P* < .0001) in the high‐risk group.

**Conclusion:**

The DSPMF is a reliable prognostic tool for survival risk in patients with NPC and might be able to guide the treatment decision.

## Graphical abstract

The research of this paper is time‐effective, innovative, and practical. It may help clinician to judge the prognosis of the patients with NPC and guide the treatment decision.

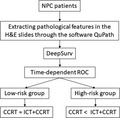

## 1 INTRODUCTION

Nasopharyngeal carcinoma (NPC) has obvious differences in regional distribution: the age‐standardized incidence rates are much higher in south China than in white populations.^1^, ^2^ Radiotherapy is the primary treatment method for nondisseminated NPC because of its concealed anatomical location and sensitivity to irradiation. Great progress has been made in treating NPC because of the development of intensity‐modulated radiation therapy (IMRT) and the use in combination with chemotherapy, but the major cause of treatment failure remains distant metastasis.^3^

Current treatment decisions and the prognosis of NPC depend mainly on the TNM staging system. Although patients with NPC receive similar treatment at the same stages, their outcomes can be different. Epstein‐Barr virus (EBV) DNA has been shown to be an effective prognostic marker and can be used to help guide treatment decisions for NPC.^4^, ^5^ In 2015, Tang et al^6^ established and validated prognostic nomograms could predict prognosis in endemic NPC. Meanwhile, many scholars thought that biological heterogeneity among other possible explanations such as viral and environmental influences may lead to differences in prognosis. In 2012, Liu et al^7^ showed that the micro‐RNA signature might inform treatment choices for patients in high risk for progression. In 2016, Tang et al^8^ developed and validated a gene‐based nomogram to predict distant metastasis of loco‐regionally advanced (LA)‐NPC.

Since 1889, when Stephen Paget put forward the theory of "seed‐soil",^9^ more and more evidence has indicated that cancer metastasis requires the coordination of tumor cells and the microenvironment. For example, chronic inflammation can promote tumorigenesis,^10^ the host immune system is equally capable of controlling tumor growth by activating adaptive and innate immune mechanisms,^11^ and so on. Many studies have shown that the microenvironment can be a prognosticator in solid tumors, including colon cancer,^12^ breast cancer,^13^ and ovarian cancer.^14^ Therefore, the emergence of digital pathology has made it feasible for us to study the microenvironment of tumors. With the advance in technologies, more and more tools have been developed and introduced to digitize pathological results, including Image J,^15^ CellProfiler,^16^ SlideToolKit,^17^ and QuPath,^18^ allowing us to analyze pathological slides automatically.

The possibility of digitizing whole slide images (WSI) of tissue has led to the advent of artificial intelligence (AI) in digital pathology, which might ultimately improve patient management.^19^ In image recognition, tremendous progress has been made because of the availability of large‐scale annotated datasets (ie ImageNet^20^) and the deep convolutional neural networks.^21^ In this study, we aimed to use the neural networks combined with the pathological microscopic features extracted by the software QuPath to assess survival risk in patients with NPC and to help make treatment decisions.

## 2 MATERIALS AND METHODS

### 2.1 Study design and ethical considerations

This retrospective clinical research enrolled patients with NPC at Sun Yat‐Sen University Cancer Center (SYSUCC). The ethics committee of the Chinese Clinical Trial Registry (ChiECRCT20190034) reviewed and approved the study protocol. All included patients had provided signed informed consent for their data to be reviewed for the later study.

### 2.2 Clinical specimens

We collected pathological slides in accordance with the regulations of the Departments of Pathology at both SYSUCC. Hematoxylin‐eosin (H&E)–stained slides of 1229 patients with NPC were collected from the Department of Pathology at SYSUCC (Guangzhou), 1055 passed quality control finally. The slides were divided into two groups using computer‐generated random numbers: 843 in the training group and 212 in the validation group. We restaged all patients based on the 8th edition of the American Joint Committee on Cancer/Union for International Cancer Control (AJCC/UICC) Staging system through magnetic resonance imaging (MRI) before the initial treatment.

All patients had received radical IMRT. Recommended radiotherapy dose was 2.13 Gy‐2.33 Gy per fraction administered daily from Monday to Friday every week for 6‐7 weeks. The total prescribed dose was 68‐70 Gy, 62‐68 Gy, 60 Gy, and 54 Gy, 30‐32 fractions in total, according to the planned target volume of GTVnx, GTVnd, CTV1, and CTV2, respectively. Specifically, GTVnx consisted of the sum of the primary tumor and the enlarged retropharyngeal nodes, and GTVnd included the volume of clinically involved gross cervical lymph nodes. CTV1, the high‐risk clinical target volume, was defined as the volume of GTVnx plus a 5‐10 mm margin to encompass the high‐risk sites of the microscopic extension and the whole nasopharynx. Low‐risk clinical target volume (CTV2) covered the volume of CTV1 plus a 5‐10 mm margin to encompass the low‐risk sites of the microscopic extension.

Most of the patients was administered to cisplatin‐based chemotherapy, containing concomitant chemoradiotherapy (CCRT), induction chemotherapy (ICT), and adjuvant chemotherapy (ACT). ICT or ACT consisted of PF (cisplatin plus 5‐fluorouracil)/TPF (cisplatin with 5‐fluorouracil and taxanes)/TP (cisplatin and taxanes) regimes every 3 weeks for two or three cycles. CCRT based on cisplatin was received on weeks 1, 4, and 7 of radiotherapy, or weekly.

### 2.3 Procedures

All slides were scanned as whole slide images (WSI) using the Aperio scanner (Leica Biosystems), with magnification 40×, resolution ratio 512 × 512 pixel, and each WSI stored as format.svs.

QuPath software (version 0.1.2. Queen's University) used to analyze the WSI. Each image represented one patient and 429 microfeatures were extracted from each H&E image. We trained 900 cells to establish a cell classifier using the automated random tree method. The 429 features were saved as a file using the.txt format for each image. Means of cell features were then calculated and each was transformed into the.xlsx format for statistical analysis. The processes above were done in the server using the Intel Xeon Gold 6128 CPU (3.4GHz/6core/19.25MB/115W).

### 2.4 Statistical analysis

The main endpoint of this study was the 5‐year progression‐free survival (PFS), calculated from the first day of initial treatment to the date of locoregional failure, or the date of distant metastasis or death from any cause, whichever occurred first. Secondary endpoints were the 5‐year distant metastasis‐free survival (DMFS), local recurrence‐free survival (LRFS), and the overall survival (OS).

We did the random hyperparameter optimization search and used k‐means cross‐validation (k = 5) to evaluate the performance of the configuration. In order to avoid models that overfit, we then choose the configuration with the largest validation C‐index. Risk values were constructed by the methods DeepSurv,^22^ a state‐of‐the‐art survival method in order to provide personalized treatment choices for modeling interactions between a patient's covariates and treatment effectiveness based on Cox proportional hazards deep neural network. We used the time‐dependent receiver‐operator characteristic (ROC) curve to find the optimal cutoff value which separated patients into two groups (high‐risk group and low‐risk group). Statistically significant variables in the univariate analyses were entered into multivariable Cox regression analysis. The independent significance of different clinical factors were tested by the multivariate Cox regression analysis, of which the P value larger than 0.05 was removed from the analysis.^8^ Kaplan‐Meier method was used to calculate time‐to‐event data, and 95% confidence interval (CI) was calculated using Greenwood's formula.

Statistical analyses were executed using in R (version 3.6.1, ://www.r-project.org/). All statistical tests were two‐sided, and *P* values of <.05 were considered significant.

## 3 RESULTS

**Figure 1:**
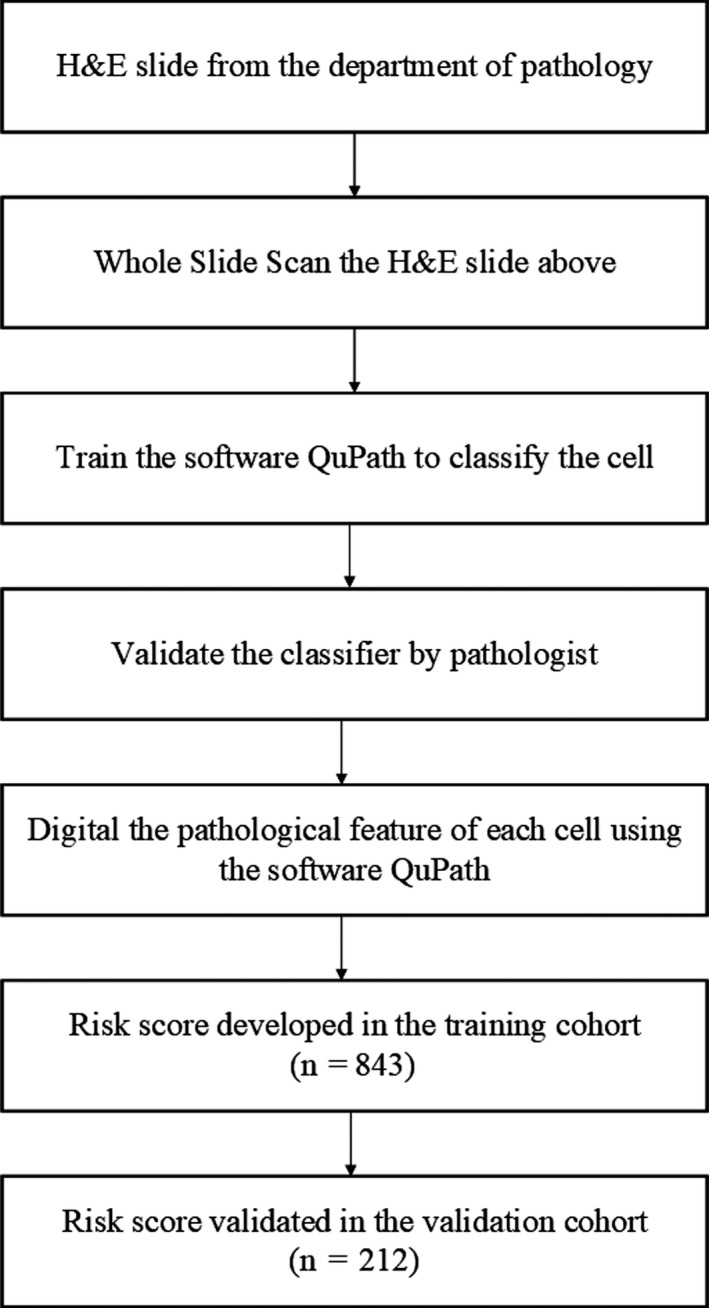
Study flow. H&E, hematoxylin and eosin

Patients’ demographic data and baseline clinical characteristics are shown in Table 1. Results of QuPath software analysis of 429 microfeatures from the H&E slides of NPC patients are displayed in the Supplemental Methods. Figure 1 shows the study flow, and the process of extracting the 143 features using the software QuPath is shown in the Supplemental Methods.

**Table 1:** The baseline and clinical characteristics

	Guangzhou training cohort			Guangzhou validation cohort		
	Patients (n = 843)	Low risk (n = 716)	High risk (n = 127)	Patients (n = 212)	Low risk (n = 172)	High risk (n = 40)
Age (y)						
<45	365	311 (85.2%)	54 (14.8%)	101	81 (80.2%)	20 (19.8%)
≥45	478	405 (84.7%)	73 (15.3%)	111	91 (82.0%)	20 (18.0%)
Sex						
Male	628	528 (84.1%)	100 (15.9%)	151	120 (79.5%)	31 (20.5%)
Female	215	188 (87.4%)	27 (12.6%)	61	52 (85.2%)	9 (14.8%)
T stage						
T1	89	77 (86.5%)	12 (13.5%)	28	24 (85.7%)	4 (14.3%)
T2	133	116 (87.2%)	17 (12.8%)	21	15 (71.4%)	6 (28.6%)
T3	398	351 (88.2%)	47 (11.8%)	109	90 (82.6%)	19 (17.4%)
T4	223	172 (77.1%)	51 (22.9%)	54	43 (79.6%)	11 (20.4%)
N stage						
N0	89	82 (92.1%)	7 (7.9%)	21	18 (85.7%)	3 (14.3%)
N1	326	291 (89.3%)	35 (10.7%)	88	74 (84.1%)	14 (15.9%)
N2	276	220 (79.7%)	56 (20.3%)	63	52 (82.5%)	11 (17.5%)
N3	152	123 (80.9%)	29 (19.1%)	40	28 (70.0%)	12 (30.0%)
TNM stage						
I	22	20 (90.9%)	2 (9.1%)	8	6 (75.0%)	2 (25.0%)
II	110	100 (90.9%)	10 (9.1%)	19	16 (84.2%)	3 (15.8%)
III	369	324 (87.8%)	45 (12.2%)	100	85 (85.0%)	15 (15.0%)
IV	342	272 (79.5%)	70 (20.5%)	85	65 (76.5%)	20 (23.5%)
EBVDNA copies						
<1000	326	288 (88.3%)	38 (11.7%)	96	76 (79.2%)	20 (20.8%)
1000‐9999	229	203 (88.6%)	26 (11.4%)	54	49 (90.7%)	5 (9.3%)
10 000‐99 999	206	162 (78.6%)	44 (21.4%)	44	32 (72.7%)	12 (27.3%)
100 000‐999 999	72	55 (76.4%)	17 (23.6%)	15	13 (86.7%)	2 (13.3%)
>1 000 000	10	8 (80.0%)	2 (20.0%)	3	2 (66.7%)	1 (33.3%)
Hemoglobin concentration (g/L)						
<120	51	43 (84.3%)	8 (15.7%)	7	6 (85.7%)	1 (14.3%)
≥120	792	673 (85.0%)	119 (15.0%)	205	166 (81.0%)	39 (19.0%)
LDH concentration（U/L）						
＜245	753	645 (85.7%)	108 (14.3%)	185	149 (80.5%)	36 (19.5%)
≥245	90	71 (78.9%)	19 (21.1%)	27	23 (85.2%)	4 (14.8%)
Treatment method						
RT alone	67	61 (91.0%)	6 (9.0%)	0	0	0
CCRT	279	222 (79.6%)	57 (20.4%)	55	38 (69.1%)	17 (30.9%)
ICT + CCRT	492	428 (87.0%)	64 (13.0%)	137	118 (86.1%)	19 (13.9%)
CCRT + ACT	5	5 (100%)	0 (0)	20	16 (80.0%)	4 (20.0%)

Abbreviations: ACT, adjuvant chemotherapy; CCRT, concurrent chemoradiotherapy; EBVDNA, Epstein‐Barr virus DNA; ICT, induction chemotherapy; LDH, serum lactate dehydrogenase levels; RT, radiotherapy.

### 3.1 Associations between pathological microfeatures and PFS

**Figure 2:**
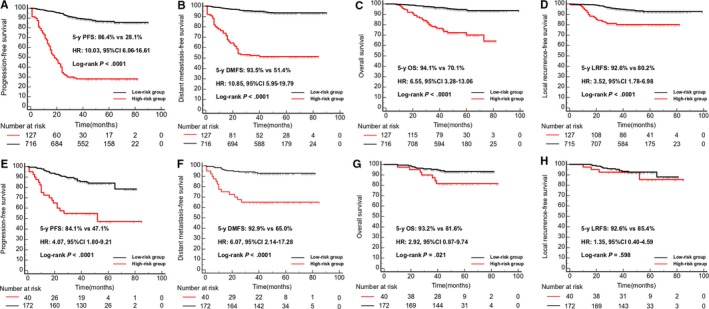
Kaplan‐Meier curves of survival analysis in the training cohort and validation cohort. A, 5‐y PFS in the training cohort, (B) 5‐year DMFS in the training cohort, (C) 5‐year OS in the training cohort, (D) 5‐year LRFS in the training cohort, (E) 5‐year PFS in the validation cohort, (F) 5‐year DMFS in the validation cohort, (G) 5‐year OS in the validation cohort, (H) 5‐year LRFS in the validation cohort. DMFS, distant metastasis‐free survival; HR, hazard ratio, CI, confidence interval.; LRFS, local recurrence‐free Survival; OS, overall survival; PFS, progression‐free survival

Risk scores were calculated for each patient using the methods DeepSurv. Patients were then divided into a high‐risk group and a low‐risk group according to the ROC curves (cutoff value = −0.563). A total of 127 of 843 patients from the training group were assigned to the high‐risk group. In the training cohort, there were 43.0% (184/428) patients complete the 3‐course concurrent chemotherapy in the low‐risk group and 40.6% (26/64) patients completed (*P* = .721, chi‐square test) in the high‐risk group. Patients in the high‐risk group had worse 5‐year PFS than in the low‐risk group, with 5‐year PFS of 28.1% and 86.4%, respectively (HR 10.03, 95% CI 6.06‐16.61, *P* < .0001, Figure 2A). The 5‐year DMFS, LRFS, and OS were also significantly different between the two groups (Figure 2B‐D).

In the Guangzhou test cohort, the DSPMF categorized 40 of 212 patients into the high‐risk group using the same cutoff value (−0.563). In the testing cohort, 48 patients in the low‐risk group and 8 patients in the high‐risk group completed 3 cycles concurrent chemotherapy (*P* = .907, chi‐square test), respectively. Five‐year PFS in the high‐risk group was also worse than the low‐risk group (HR 4.07, 95% CI 1.80‐9.21; *P* < .0001, Figure 2E). The 5‐year DMFS and OS were also significantly different between the two groups (Figure 2F‐G). Because the number of patients suffered from local recurrence was small, the 5‐year LRFS did not have significant difference.

### 3.2 DSPMF is an independent prognostic risk factor

**Figure 3:**
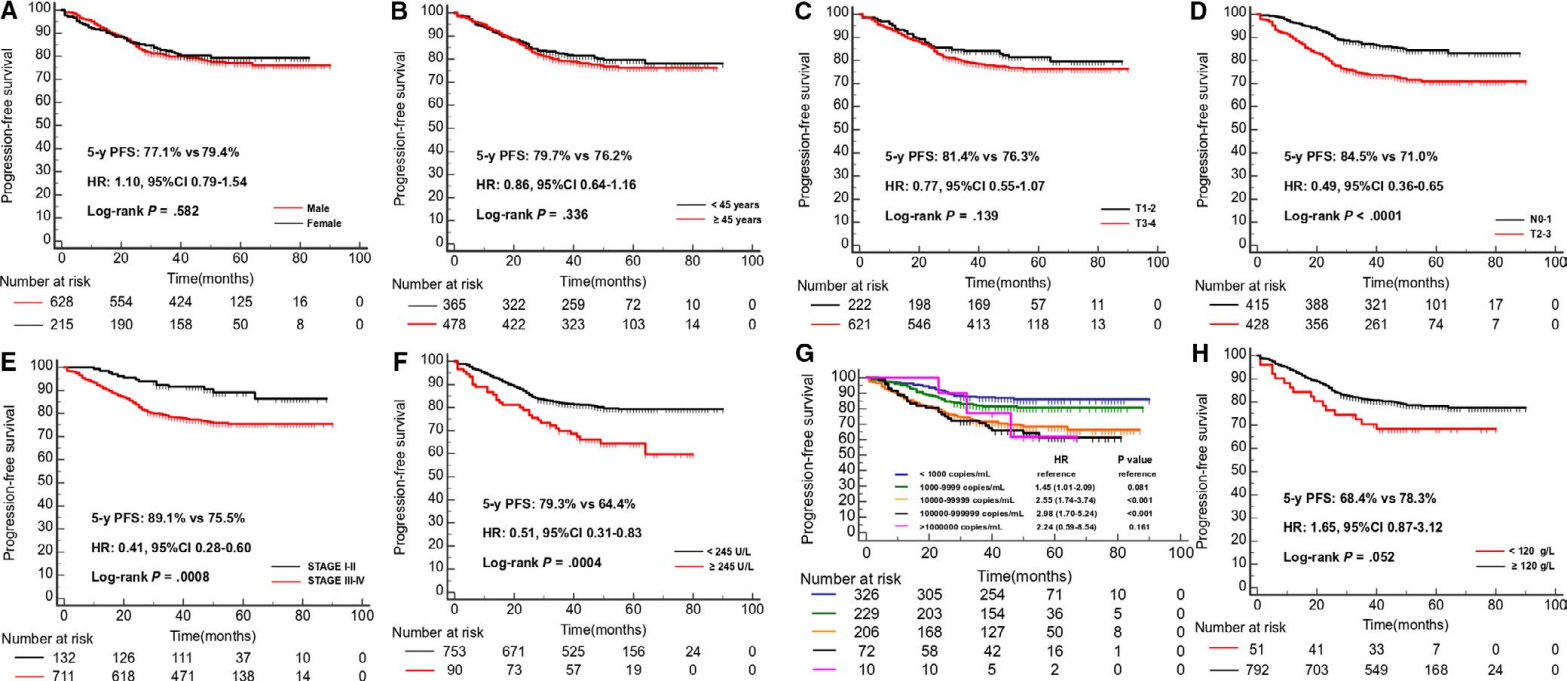
Kaplan‐Meier curves of survival analysis about the clinical variables in the training cohort. A, 5‐year PFS of the male vs the female in the training cohort; (B) 5‐year PFS of the patients less than 45 years old vs more than 45 years old in the training cohort; (C) 5‐year PFS of patients with T1‐2 vs T3‐4 in the training cohort; (D) 5‐year PFS of patients with N0‐1 vs N2‐3 in the training cohort; (E) 5‐year PFS of patients with STAGE I‐II vs STAGE III‐IV in the training cohort; (F) 5‐year PFS of patients with LDH less than 245U/L vs LDH more than 245U/L in the training cohort; (G) 5‐year PFS of patients with EBVDNA (every 10‐fold increase)in the training cohort; (H) 5‐year PFS of patients with HGB less than 120g/L vs HGB more than 120 g/L in the training cohort. HGB, hemoglobin concentration; HR, hazard ratio, CI, confidence interval.; LDH, serum lactate dehydrogenase levels; PFS, progression‐free survival

We also did univariate analysis about the clinical variables based on some studies, the variable included gender, age (<45 years vs ≥45 years), the primary tumor (T) stage (T1‐2 vs T3‐4), regional lymph nodes (N) stage (N0‐1 vs N2‐3), TNM stage (Stage I‐II vs Stage III‐IV), serum lactate dehydrogenase (LDH) (≥245 vs <245 U/L), hemoglobin (HGB) (<120 vs ≥120 g/L), and EBV DNA (every 10‐fold increase).^6^ Results of univariate analysis showed that N stage, TNM stage, EBV DNA and LDH were significantly associated with 5‐year PFS (Figure 3). We did a multivariate analysis which the covariates included the EBV DNA, LDH, N stage, TNM stage, and DSPMF. Results showed that the EBV DNA, LDH, and DSPMF are the independent prognostic risk factors for 5‐year PFS in patients with NPC (Table. 2).

**Table 2:** Univariate and multivariate analysis for 5‐year PFS in the Guangzhou training cohort

Variable	HR (95%CI)	*P* value	C‐index
N stage		.083	0.593
N0‐1	Reference		
N2‐3	2.05 (1.53‐2.75)		
TNM stage		.133	0.551
I‐II	Reference		
III‐IV	2.45 (1.66‐3.61)		
EBVDNA (copy/mL)		.019	0.612
<1000	Reference		
1000‐9999	1.45 (0.94‐2.23)		
10 000‐99 999	2.54 (1.72‐3.76)		
100 000‐999 999	2.99 (1.59‐5.62)		
>1 000 000	2.25 (0.41‐12.25)		
LDH（U/L）		.008	0.538
<245	Reference		
≥245	1.96 (1.20‐3.19)		
Risk group		<.001	0.723
Low‐risk	Reference		
High‐risk	10.03 (6.06‐16.61)		

Abbreviations: CI, confidence interval; EBVDNA, Epstein‐Barr virus DNA; HGB, hemoglobin; HR, hazard ratio; LDH, serum lactate dehydrogenase levels

### 3.3 DSPMF associated with treatment decisions

**Figure 4:**
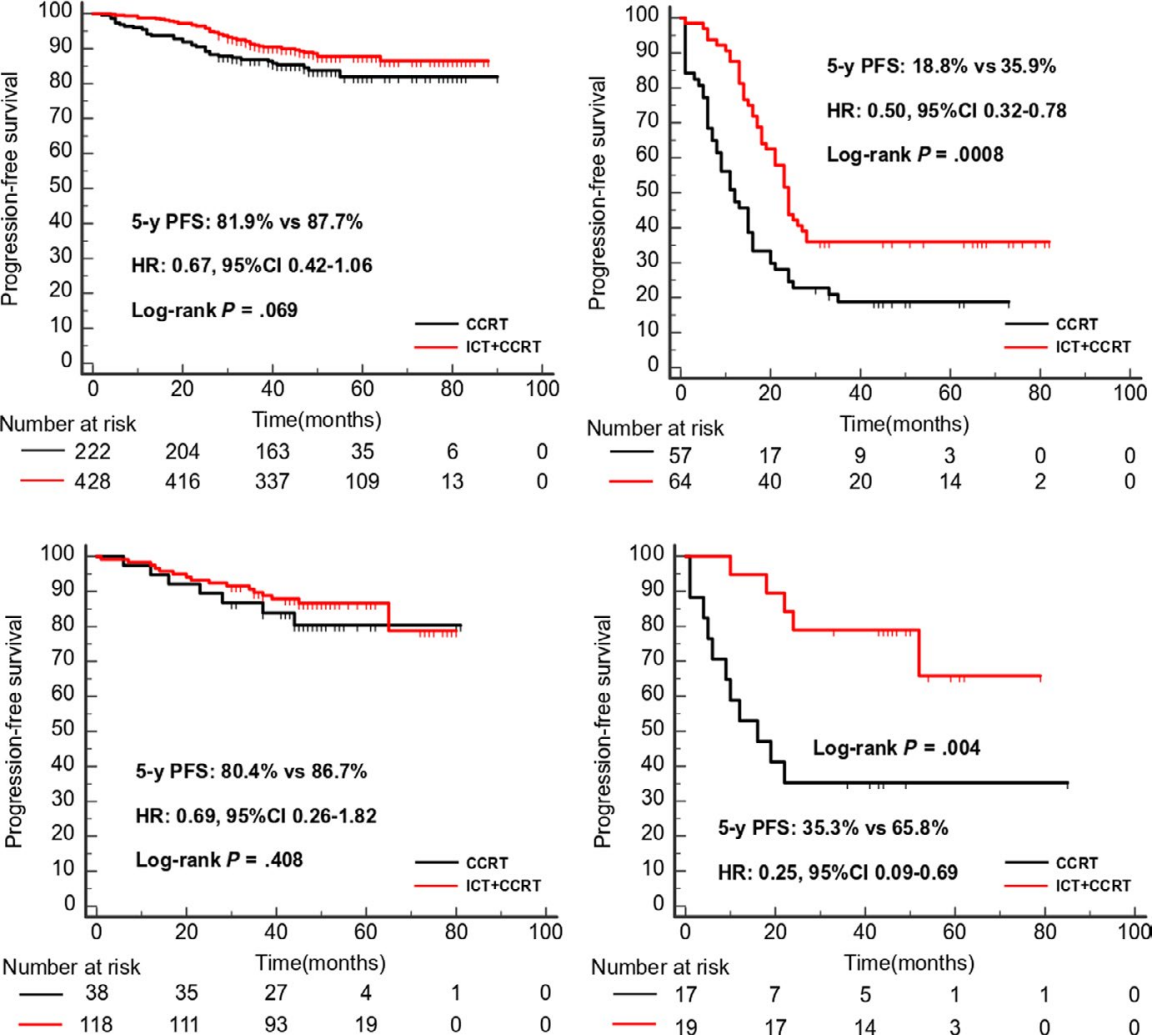
Kaplan‐Meier curves of survival analysis of 5‐year PFS for patients received ICT + CCRT vs CCRT alone. A, The patients of low risk in the training cohorts; (B) The patients of high risk in the training cohorts; (C) The patients of low risk in the validation cohorts; (D) The patients of high risk in the validation cohorts

In the Guangzhou training cohort, in the low‐risk group 222 patients received concurrent chemoradiotherapy (CCRT) alone vs 428 patients received induction chemotherapy (ICT)+CCRT and in the high‐risk group 57 patients received CCRT alone vs 64 patients received ICT + CCRT. The 5‐year PFS between ICT + CCRT and CCRT in the low‐risk group was similar (HR 0.67, 95%CI:0.42‐1.06, *P* = .069; Figure 4A), whereas in the high‐risk group, patients who received ICT + CCRT had longer 5‐year PFS than CCRT alone (HR 0.50, 95%CI 0.32‐0.78, *P* = .0008, Figure 4B). These outcomes were validated in the validation cohort. (Figure 4C‐D**)**.

## 4 DISCUSSION

As far as we know, this is the first research to predict patients’ survival risk in NPC combined pathological microfeatures with deep learning. When we used the validated DSPMF to categorize patients into two groups (high‐risk group and low‐risk group), results showed significantly different survival risks for PFS, DMFS, and OS between the two groups.

In many solid tumors, the pathological features have been shown to be an effective tool with which to predict survival risk associated with various disease states.^13^, ^23^, ^24^, ^25^ The 149 features included count ratio, nucleus perimeter, and Nucleus: Hematoxylin OD. About the count ratio, Ma et al^26^ found that tumor‐infiltrating lymphocytes (TILs) were independent prognostic indicators for disease‐free survival (DFS) in NPC. Pathologists have observed that NPC tumor cells have obvious morphologic variations, with cells that are small and round, large and round, spindle‐shaped, with or without vesicular nuclei, or mixed round and spindle‐shaped. The tumor cell with vesicular nuclei had lower value of optical density than that without vesicular nuclei. Owing to this, Shao et al^27^ proposed a new NPC histopathologic classification that can potentially be used to predict treatment response and prognosis. Combined the study of Shao, the Nucleus: Hematoxylin OD may be negatively correlated with the prognosis of patients with NPC. Whether these characteristics are related to some genes or other biological information remains to be further studied.

In digital pathology, artificial intelligence approaches have been applied to many image processing, such as detection,^28^, ^29^ segmentation,^30^ predicting disease diagnosis, and prognosis of treatment response on the basis of patterns in the image.^31^, ^32^ Our study combined the artificial intelligence with digital pathology to reveal the prognostic value of pathological microscopic features in patients with NPC. It helped us identify patients with poor prognosis. This research had obvious innovation and practical. What's more, it was easy to operate, did not add an additional burden to patients and took less than one minute to complete the analysis of one slide in a computer using the Intel Xeon Gold 6128 CPU (3.4GHz/6core/19.25MB/115W).

Our results also showed that people in the low‐risk group had similar results whether they received CCRT or ICT + CCRT. At the same time, patients in the high‐risk group who received ICT + CCRT had better survival than those receiving CCRT. Because ICT increased both treatment time and the occurrence of side effects, many scholars had wanted to find a method of determining which patients were most likely to benefit from receiving ICT.^33^ Therefore, our study may offer a reference to support that CCRT alone may be sufficient for patients in the low‐risk group. Meanwhile, several phase II/III studies have shown encouraging outcomes associated with ICT in patients with LA‐NPC.^34^, ^35^, ^36^, ^37^, ^38^, ^39^, ^40^ Also, 2019 NCCN guidelines have changed the category recommendation for ICT followed by chemoradiotherapy from category 3 to category 2A (non‐EBV DNA–associated) and category 1 (EBV DNA–associated), indicating that patients in the high‐risk group should receive ICT + CCRT.

Our study had some limitations. First, all data evaluated retrospectively confused making any inferences about causality and might limit generalizations to other centers or populations. Second, this study only consisted of undifferentiated non‐keratinization NPC in the endemic regions, and whether it fitted other pathological patterns and other regions was unknown. Third, we only found that the pathological microfeatures help to guide treatment, but its principles were not clear.

In summary, the DSPMF is a reliable to estimate survival risk in patients with NPC and may be useful in predicting whether patients would benefit from ICT. The results of this study may help to guide treatment decisions for patients with NPC.

## CONFLICT OF INTEREST

The authors have no conflict of interest.

## Data Availability

The data are not available for public access because of patient privacy concerns but are available from the corresponding author on reasonable request approved by the institutional review boards of Sun Yat‐sen University Cancer Center.
