# Supplementary material for: Deep learning pathological microscopic features in endemic nasopharyngeal cancer: Prognostic value and protentional role for individual induction chemotherapy
Source: Cancer Med. 2019 Dec 20;9(4):1298–306. doi: 10.1002/cam4.2802 (PMC7013063; doi:10.1002/cam4.2802)
Supplement: Supplementary file 1 [file CAM4-9-1298-s001.docx]

**Methods**

**143 Pathological micro features by cell type**

1 count ratio

2 Area ratio

3 Nucleus Area

4 Nucleus Perimeter

5 Nucleus Circularity

6 Nucleus Max caliper

7 Nucleus Min caliper

8 Nucleus Eccentricity

9 Nucleus Hematoxylin OD mean

10 Nucleus Hematoxylin OD sum

11 Nucleus Hematoxylin OD std dev

12 Nucleus Hematoxylin OD max

13 Nucleus Hematoxylin OD min

14 Nucleus Hematoxylin OD range

15 Nucleus Eosin OD mean

16 Nucleus Eosin OD sum

17 Nucleus Eosin OD std dev

18 Nucleus Eosin OD max

19 Nucleus Eosin OD min

20 Nucleus Eosin OD range

21 Cell Area

22 Cell Perimeter

23 Cell Circularity

24 Cell Max caliper

25 Cell Min caliper

26 Cell Eccentricity

27 Cell Eosin OD mean

28 Cell Eosin OD std dev

29 Cell Eosin OD max

30 Cell Eosin OD min

31 Cytoplasm Eosin OD mean

32 Cytoplasm Eosin OD std dev

33 Cytoplasm Eosin OD max

34 Cytoplasm Eosin OD min

35 Nucleus/Cell area ratio

36 Hematoxylin (25.0 mm) Mean

37 Hematoxylin (25.0 mm) Min

38 Hematoxylin (25.0 mm) Max

39 Hematoxylin (25.0 mm) Range

40 Hematoxylin (25.0 mm) Std.dev.

41 Hematoxylin (25.0 mm) coherence

42 Eosin (25.0 mm) Mean

43 Eosin (25.0 mm) Min

44 Eosin (25.0 mm) Max

45 Eosin (25.0 mm) Range

46 Eosin (25.0 mm) Std.dev.

47 Eosin (25.0 mm) coherence

48 Smoothed 25 mm Nucleus Area

49 Smoothed 25 mm Nucleus Perimeter

50 Smoothed 25 mm Nucleus Circularity

51 Smoothed 25 mm Nucleus Max caliper

52 Smoothed 25 mm Nucleus Min caliper

53 Smoothed 25 mm Nucleus Eccentricity

54 Smoothed 25 mm Nucleus Hematoxylin OD mean

55 Smoothed 25 mm Nucleus Hematoxylin OD sum

56 Smoothed 25 mm Nucleus Hematoxylin OD std dev

57 Smoothed 25 mm Nucleus Hematoxylin OD max

58 Smoothed 25 mm Nucleus Hematoxylin OD min

59 Smoothed 25 mm Nucleus Hematoxylin OD range

60 Smoothed 25 mm Nucleus Eosin OD mean

61 Smoothed 25 mm Nucleus Eosin OD sum

62 Smoothed 25 mm Nucleus Eosin OD std dev

63 Smoothed 25 mm Nucleus Eosin OD max

64 Smoothed 25 mm Nucleus Eosin OD min

65 Smoothed 25 mm Nucleus Eosin OD range

66 Smoothed 25 mm Cell Area

67 Smoothed 25 mm Cell Perimeter

68 Smoothed 25 mm Cell Circularity

69 Smoothed 25 mm Cell Max caliper

70 Smoothed 25 mm Cell Min caliper

71 Smoothed 25 mm Cell Eccentricity

72 Smoothed 25 mm Cell Eosin OD mean

73 Smoothed 25 mm Cell Eosin OD std dev

74 Smoothed 25 mm Cell Eosin OD max

75 Smoothed 25 mm Cell Eosin OD min

76 Smoothed 25 mm Cytoplasm Eosin OD mean

77 Smoothed 25 mm Cytoplasm Eosin OD std dev

78 Smoothed 25 mm Cytoplasm Eosin OD max

79 Smoothed 25 mm Cytoplasm Eosin OD min

80 Smoothed 25 mm Nucleus/Cell area ratio

81 Smoothed 25 mm Hematoxylin (25.0 mm) Mean

82 Smoothed 25 mm Hematoxylin (25.0 mm) Min

83 Smoothed 25 mm Hematoxylin (25.0 mm) Max

84 Smoothed 25 mm Hematoxylin (25.0 mm) Range

85 Smoothed 25 mm Hematoxylin (25.0 mm) Std.dev.

86 Smoothed 25 mm Hematoxylin (25.0 mm) coherence

87 Smoothed 25 mm Eosin (25.0 mm) Mean

88 Smoothed 25 mm Eosin (25.0 mm) Min

89 Smoothed 25 mm Eosin (25.0 mm) Max

90 Smoothed 25 mm Eosin (25.0 mm) Range

91 Smoothed 25 mm Eosin (25.0 mm) Std.dev.

92 Smoothed 25 mm Eosin (25.0 mm) coherence

93 Smoothed 25 mm Nearby detection counts

94 Nucleus Shape Area mm^2

95 Cell Shape Area mm^2

96 Smoothed 20 mm Nucleus Area

97 Smoothed 20 mm Nucleus Perimeter

98 Smoothed 20 mm Nucleus Circularity

99 Smoothed 20 mm Nucleus Max caliper

100 Smoothed 20 mm Nucleus Min caliper

101 Smoothed 20 mm Nucleus Eccentricity

102 Smoothed 20 mm Nucleus Hematoxylin OD mean

103 Smoothed 20 mm Nucleus Hematoxylin OD sum

104 Smoothed 20 mm Nucleus Hematoxylin OD std dev

105 Smoothed 20 mm Nucleus Hematoxylin OD max

106 Smoothed 20 mm Nucleus Hematoxylin OD min

107 Smoothed 20 mm Nucleus Hematoxylin OD range

108 Smoothed 20 mm Nucleus Eosin OD mean

109 Smoothed 20 mm Nucleus Eosin OD sum

110 Smoothed 20 mm Nucleus Eosin OD std dev

111 Smoothed 20 mm Nucleus Eosin OD max

112 Smoothed 20 mm Nucleus Eosin OD min

113 Smoothed 20 mm Nucleus Eosin OD range

114 Smoothed 20 mm Cell Area

115 Smoothed 20 mm Cell Perimeter

116 Smoothed 20 mm Cell Circularity

117 Smoothed 20 mm Cell Max caliper

118 Smoothed 20 mm Cell Min caliper

119 Smoothed 20 mm Cell Eccentricity

120 Smoothed 20 mm Cell Eosin OD mean

121 Smoothed 20 mm Cell Eosin OD std dev

122 Smoothed 20 mm Cell Eosin OD max

123 Smoothed 20 mm Cell Eosin OD min

124 Smoothed 20 mm Cytoplasm Eosin OD mean

125 Smoothed 20 mm Cytoplasm Eosin OD std dev

126 Smoothed 20 mm Cytoplasm Eosin OD max

127 Smoothed 20 mm Cytoplasm Eosin OD min

128 Smoothed 20 mm Nucleus/Cell area ratio

129 Smoothed 20 mm Hematoxylin (25.0 mm) Mean

130 Smoothed 20 mm Hematoxylin (25.0 mm) Min

131 Smoothed 20 mm Hematoxylin (25.0 mm) Max

132 Smoothed 20 mm Hematoxylin (25.0 mm) Range

133 Smoothed 20 mm Hematoxylin (25.0 mm) Std.dev.

134 Smoothed 20 mm Hematoxylin (25.0 mm) coherence

135 Smoothed 20 mm Eosin (25.0 mm) Mean

136 Smoothed 20 mm Eosin (25.0 mm) Min

137 Smoothed 20 mm Eosin (25.0 mm) Max

138 Smoothed 20 mm Eosin (25.0 mm) Range

139 Smoothed 20 mm Eosin (25.0 mm) Std.dev.

140 Smoothed 20 mm Eosin (25.0 mm) coherence

141 Smoothed 20 mm Nucleus Shape Area mm^2

142 Smoothed 20 mm Cell Shape Area mm^2

143 Smoothed 20 mm Nearby detection counts

**The process of obtaining 143 features using the software QuPath**

The processes of extract features are as below: (1) Estimate stain vectors; (2) Simple tissue detection; (3) Create tiles; (4) Cell detection; (5) Calculate features; (6) Develop detection classifier (Random trees). Script of extraction of digital pathology micro features by QuPath software, as follows:

**setImageType** ('BRIGHTFIELD_H_E');

**setColorDeconvolutionStains** ('{"Name": "H&E default", "Stain 1": "Hematoxylin", "Values 1": "0.6652 0.68965 0.28615 ", "Stain 2": "Eosin", "Values 2": "0.51767 0.76119 0.39064 ", "Background": " 184 135 188 "}');

**runPlugin**('qupath.imagej.detect.tissue.SimpleTissueDetection2','{"threshold": 225,"requestedPixelSizeMicrons":20.0,"minAreaMicrons":100000.0,"maxHoleAreaMicrons":1000000.0,"darkBackground":false,"smoothImage":false,"medianCleanup":false,"dilateBoundaries":false,"smoothCoordinates": true, "excludeOnBoundary":false, "singleAnnotation": false}');

**select Annotations ()**;

**runPlugin**('qupath.imagej.detect.nuclei.WatershedCellDetection','{"detectionImageBrightfield":"HematoxylinOD","requestedPixelSizeMicrons":0.0,"backgroundRadiusMicrons":8.0,"medianRadiusMicrons":0.0,"sigmaMicrons":1.2,"minAreaMicrons":5.0,"maxAreaMicrons":400.0,"threshold":0.1,"maxBackground":2.0,"watershedPostProcess":true, "cellExpansionMicrons": 5.0, "includeNuclei": true,"smoothBoundaries": true,"makeMeasurements": true}');

**runPlugin**('qupath.lib.plugins.objects.SmoothFeaturesPlugin','{"fwhmMicrons": 25.0,"smoothWithinClasses": false, "useLegacyNames": false}');

**runPlugin**('qupath.imagej.detect.cells.SubcellularDetection','{"detection[Eosin]":1.0,"doSmoothing":false,"splitByIntensity":false,"splitByShape":false,"spotSizeMicrons":1.0,"minSpotSizeMicrons":0.5,"maxSpotSizeMicrons":2.0, "includeClusters": true}');

**runClassifier** ('D:\\cell classifier. qpclassifier');

**def name** = getProjectEntry (). getImageName () + '.txt'

**def path** = buildFilePath (PROJECT_BASE_DIR, 'detection results')

**mkdirs**(path)

**path** = buildFilePath (path, name)

**saveDetectionMeasurements**(path)
